# Reduced Satellite Cell Numbers and Myogenic Capacity in Aging Can Be Alleviated by Endurance Exercise

**DOI:** 10.1371/journal.pone.0013307

**Published:** 2010-10-12

**Authors:** Gabi Shefer, Gat Rauner, Zipora Yablonka-Reuveni, Dafna Benayahu

**Affiliations:** 1 Department of Cell and Developmental Biology, Sackler Faculty of Medicine, Tel-Aviv University, Tel-Aviv, Israel; 2 Department of Biological Structure, University of Washington School of Medicine, Seattle, Washington, United States of America; McMaster University, Canada

## Abstract

**Background:**

Muscle regeneration depends on satellite cells, myogenic stem cells that reside on the myofiber surface. Reduced numbers and/or decreased myogenic aptitude of these cells may impede proper maintenance and contribute to the age-associated decline in muscle mass and repair capacity. Endurance exercise was shown to improve muscle performance; however, the direct impact on satellite cells in aging was not yet thoroughly determined. Here, we focused on characterizing the effect of moderate-intensity endurance exercise on satellite cell, as possible means to attenuate adverse effects of aging. Young and old rats of both genders underwent 13 weeks of treadmill-running or remained sedentary.

**Methodology:**

Gastrocnemius muscles were assessed for the effect of age, gender and exercise on satellite-cell numbers and myogenic capacity. Satellite cells were identified in freshly isolated myofibers based on Pax7 immunostaining (i.e., ex-vivo). The capacity of individual myofiber-associated cells to produce myogenic progeny was determined in clonal assays (in-vitro). We show an age-associated decrease in satellite-cell numbers and in the percent of myogenic clones in old sedentary rats. Upon exercise, there was an increase in myofibers that contain higher numbers of satellite cells in both young and old rats, and an increase in the percent of myogenic clones derived from old rats. Changes at the satellite cell level in old rats were accompanied with positive effects on the lean-to-fat Gast muscle composition and on spontaneous locomotion levels. The **significance** of these data is that they suggest that the endurance exercise-mediated boost in both satellite numbers and myogenic properties may improve myofiber maintenance in aging.

## Introduction

The ability of skeletal muscles to regenerate is owed to a population of myogenic stem cells called satellite cells [1], [2], [3]. These adult stem cells are situated under the basal lamina of myofibers and contribute 2–4% of the nuclei in adult skeletal muscles [2]. Satellite cells are typically quiescent in adult muscles, but can be activated in response to muscle injury and disease. Depending on the magnitude of tissue trauma, these cells may divide minimally to repair subtle damage within individual myofibers or produce a larger progeny pool that forms new myofibers in cases of overt muscle trauma [4], [5]. Satellite cells meet the functional definition of what stem cells are, as they have the ability to self-renew, in addition to producing differentiating progeny. Clonal analyses of satellite cells suggested that satellite cells are heterogeneous with regard to their self-renew capacity and to the extent of progeny they can produce [6], [7].

The common marker used to identify satellite cells in their niche is the paired box transcription factor Pax7. As shown across different muscle groups and species, Pax7 protein is expressed by satellite cells, but not by myofiber nuclei or non-myogenic cell types present in the adult muscle tissue [8], [9], [10], [11]. Proliferating progeny of satellite cells, myoblasts, maintain Pax7 protein expression and upregulate the expression of MyoD, a muscle specific transcription factor. In differentiating myoblasts, Pax7 expression diminishes, whereas the expression of MyoD is maintained [3], [12].

In aging, skeletal muscle mass and performance decline, a process named sarcopenia [13]. Considering the key role of satellite cells in myofiber repair, diminution in their numbers and myogenic properties may impede muscle maintenance and contribute to sarcopenia. Indeed, age-associated alterations in satellite cells were reported, including increased adipogenic gene expression and diversion of at least some of the cells to a nonmyogenic (fibrogenic) fate [14], [15]. Also, satellite cell ability to contribute to muscle repair was suggested to decrease with age. This was based on rodent models in which the regenerative response of muscles was examined after an induced injury. However, the satellite cells themselves were not analyzed directly [16], [17]. Muscle damage resulting from routine activity may, however, involve only subtle damages localized to individual myofibers. In such localized injuries, the repair potential of individual myofibers may depend on the abundance of the satellite cells they harbor.

While it remains unclear if the ability of satellite cells to contribute to muscle repair is indeed impaired in old age [18], an age-associated decline in the number of satellite cells was certainly documented in mice and rats, at least in some limb muscles [19], [20], [21], [22]. We demonstrated that in mice there was an increase in myofibers with none or low numbers of satellite cells with age [6], [9]. The decline was more robust in the fast-twitch extensor digitorum longus (EDL) muscle compared to the slow-twitch soleus muscle [9], in accordance with the preferential loss and atrophy of fast twitch fibers in sarcopenia. These findings prompted us to use approaches that can provide insights about satellite-cell numbers and properties within the context of individual myofibers [9]. Taken together, in the present study we investigated the impact of exercise on the properties and number of satellite cells situated on Gastrocnemius (Gast) myofibers of intact male and female rats. Myofibers were isolated from the superficial region of the Gast muscle which was shown to contain fast-twitch myofibers (IIx or IIb) almost exclusively [23], [24], [25].

To date, the effect of endurance training on satellite cells was not thoroughly elucidated albeit this type of exercise was shown to have beneficial effects on skeletal muscle integrity even in sufferers from myopathies [26], [27], [28]. Here we chose to use endurance exercise as it was shown not to inflict apparent damage, different from resistance exercise [29], [30]. Males and females were analyzed in view of the significant gender differences in both the prevalence of sarcopenia and the extent of satellite cell decline in aging [6], [31].

Our main finding is that running exercise induces a significant increase in the abundance of myofibers with higher content of satellite cells. This was accompanied by a reduction in the abundance of myofibers that contain minimal numbers of satellite cells. Importantly, we also show that in old rats, exercise inflicted a greater proportion of myogenic clones, and this may reflect an improvement of satellite cell myogenic performance. The boost of satellite-cell numbers and myogenic performance represents a possible mechanism by which endurance exercise enhances muscle quality in old age.

## Results

### The experimental model and subsequent assays

**Figure 1:**
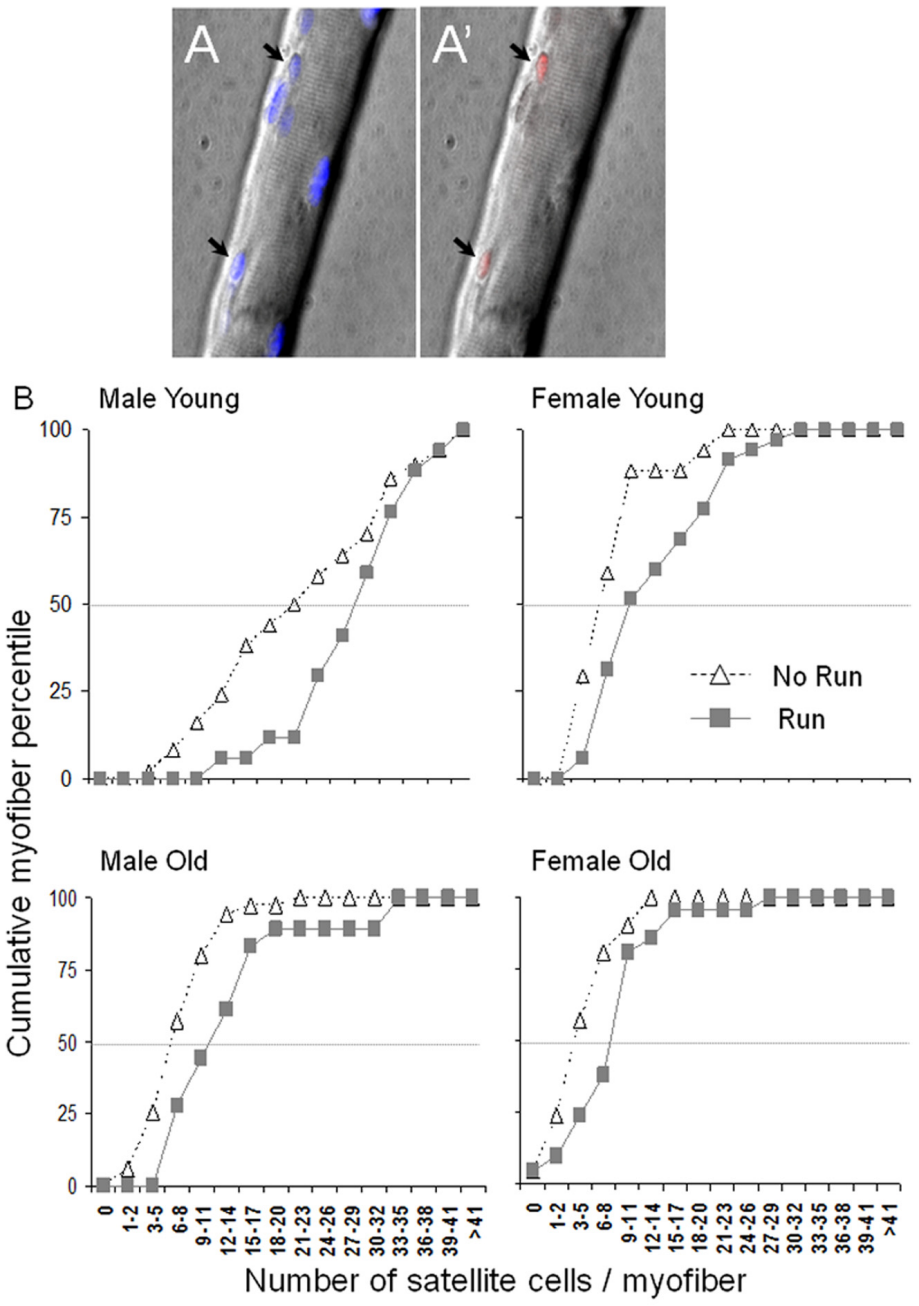
Satellite cell localization and quantification. (A) A Gast myofiber from a young exercised male rat depicting two Pax7^+^ (red) satellite cells (A′); satellite cell nuclei (arrowhead) and myofiber nuclei counterstained with DAPI (blue; A). (B) Quantification of Pax7^+^ satellite cells situated in Gast myofibers isolated from male female young old sedentary (black broken line) and exercised (gray line) rats. The number of myofibers, the minimum and maximum values of satellite cells per myofiber as well as the average number of satellite cells per myofiber for each group are detailed in Table 1. Per each experimental group, myofibers were ranked according to the number of satellite cells they contained from low to high. The y-axis represents the cumulative percentile of the analyzed myofibers. Each data point (black triangle or gray square) displays the accumulating percent of myofibers (Y axis) that contain the specific range of Pax7^+^ cells that is presented in the X axis plus all the myofibers with a lower number of satellite cells. Gray broken line depicts the median value of satellite cells per myofiber.

**Figure 2:**
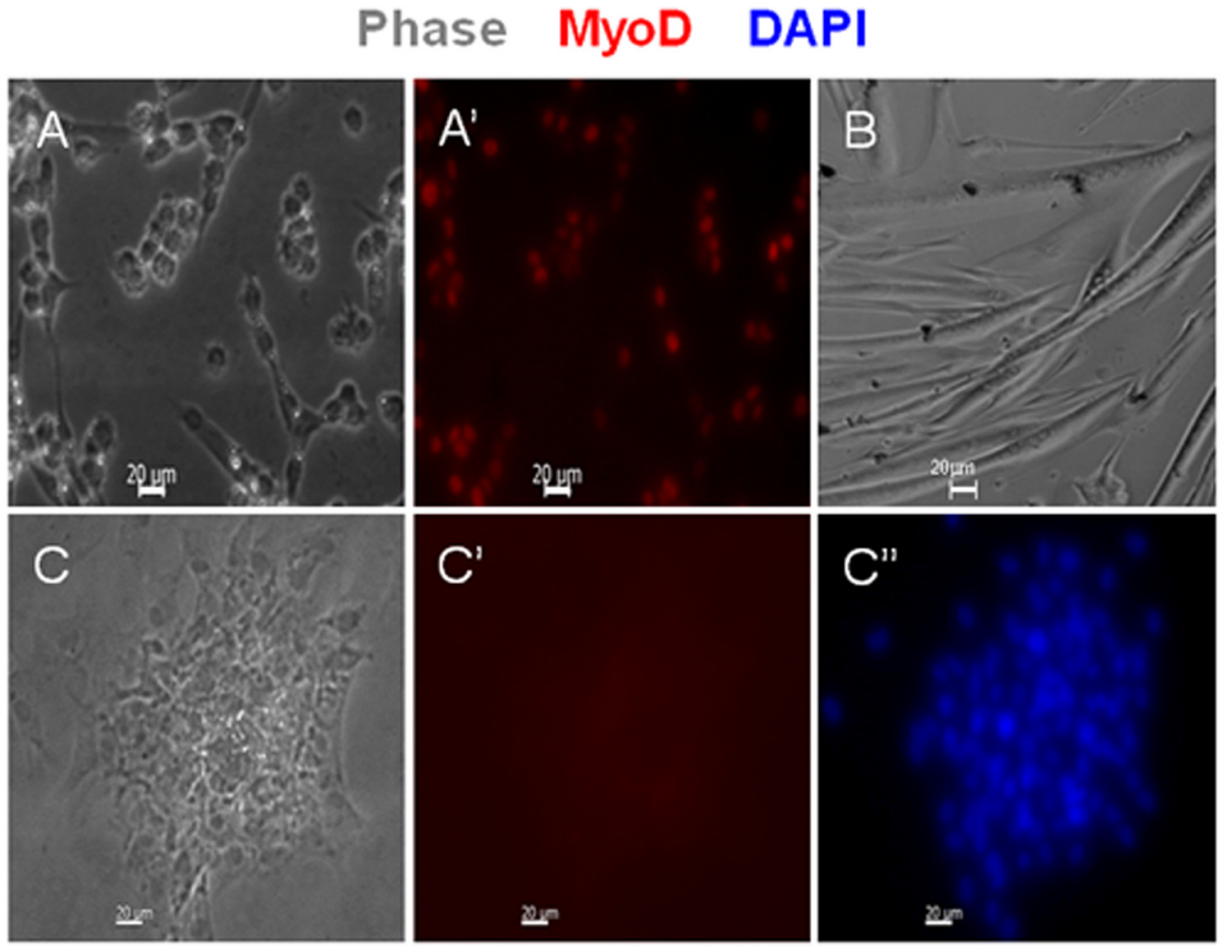
Cell morphology and expression of the skeletal muscle specific marker MyoD in myogenic and nonmyogenic clones. The myogenic clones (A, A', B) are composed of round cells and myotubes (A), both of which express MyoD (red, A'), or just myotubes (B). The nonmyogenic clone depicted in panel C is composed of fibroblast-like cells that do not express MyoD (C').

**Figure 3:**
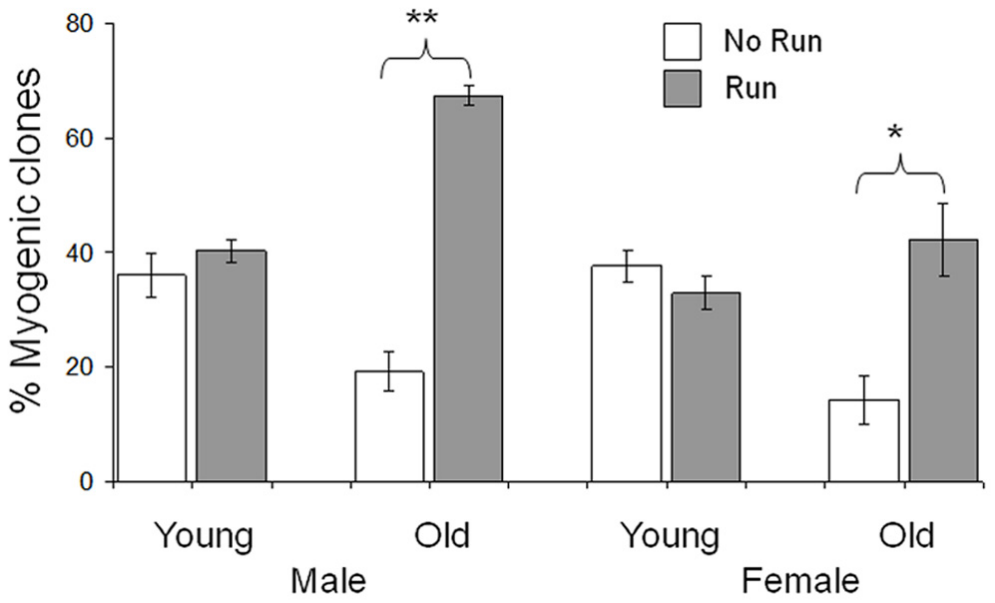
Average values of the percent of myogenic clones that developed from single cells extracted from Gast myofibers. Each bar depicts the average values of young/old, male/female exercised (open bars) and sedentary (gray bars) rats. Error bars indicate standard error of the mean.

**Figure 4:**
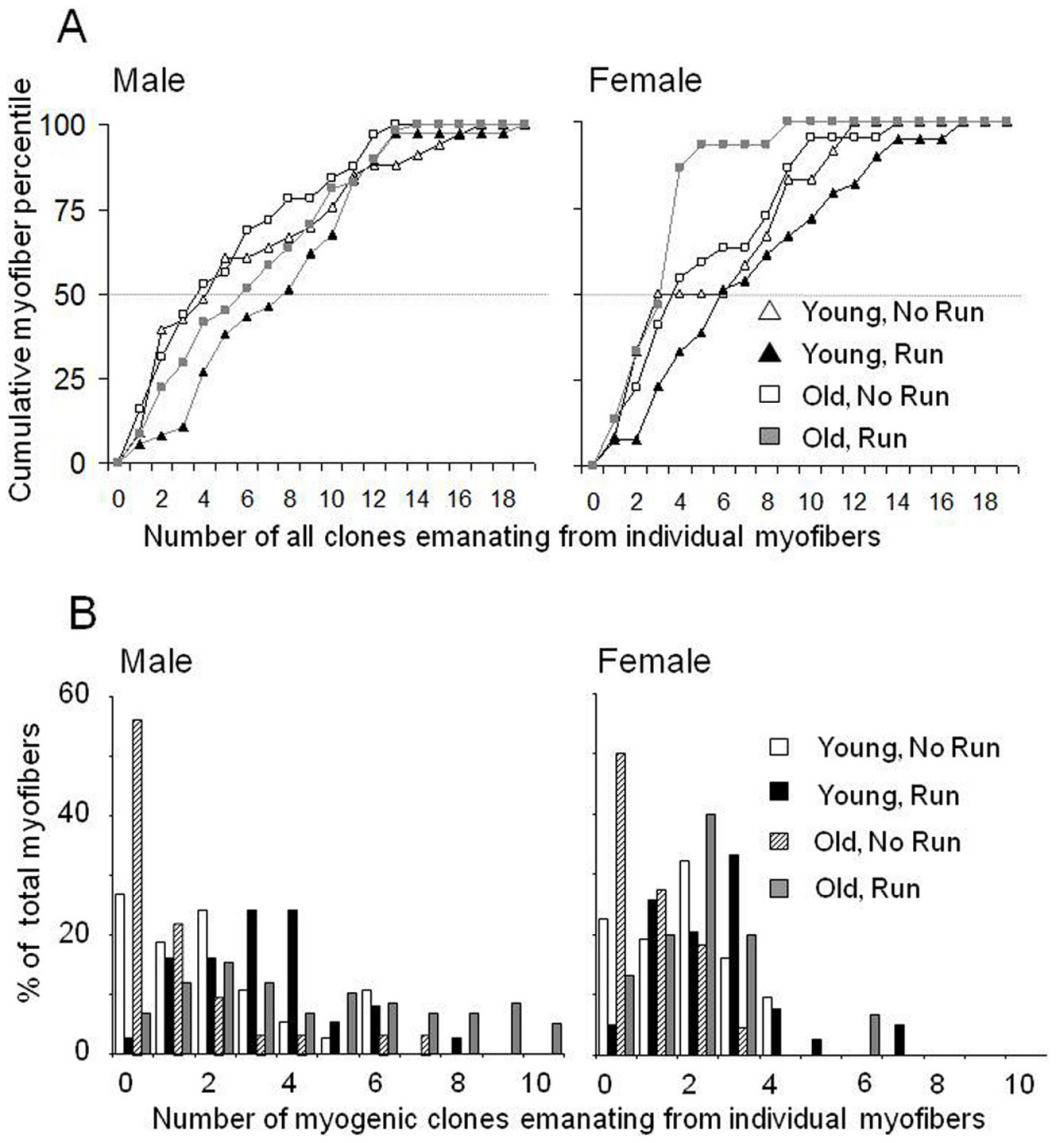
Quantification of clones (total and myogenic only) recovered from individual Gast myofibers from male, female, young, old sedentary and exercised rats. (A) Myofibers were ranked from low to high according to the total number of clones (myogenic and nonmyogenic) they gave rise-to. The y-axis represents the cumulative percentile of the analyzed myofibers. Each data point (triangle or square) represents the accumulating percent of myofibers (y axis) that contain the number of clones that is presented in the X axis in addition to all the myofibers that gave rise to lower numbers of clones. Gray broken line depicts the median (50%) value of clones per myofiber. (B) Each bar represents the percent of myofibers that gave rise to the number of myogenic clones, indicated in the X axis. Percent values for male and female rats are depicted as follows: open bard for young sedentary, black bars for young exercised, striped bars for old sedentary and gray bars for old exercised.

This study was designed to investigate the effect of long-term treadmill running on satellite cell performance in young and old male and female rats. Animals' age at the beginning of the 13 week No-Run/Run experiment were: young male/female groups, 3.5 months; old male groups, 15–17 months; old female groups, 15 months (see Table 1). For 13 weeks, animals exercised 20 minutes on a treadmill, 6 days/week followed by a day off (see a video of exercising rats, Video S1). Control groups remained sedentary for that period. At the 13-week exercise period, single myofibers were isolated from the Gast muscle and analyzed for their number of residing satellite cells (Figure 1, and Tables 1, 2). Clones were prepared from these isolated myofibers for cell phenotype studies (Figures 2, 3, 4 and Table 3). We also measured total body weight, in-vivo mass and fat content of the Gast muscle, and levels of spontaneous locomotion of all the animals in order to assess the effects of exercise at the levels of gross muscle anatomy (see Figure S1 and Figure S2).

**Table 1:** Descriptive statistics of the number of satellite cells in individual myofibers in young sedentary and exercised males and females.^1^

			Number of:		Satellite-cell numbers per myofiber:		
			Rats	Myofibers	Minimum	Maximum	Mean±SEM 2
**Male**	Young	No Run	3	49	4	48	24±2^b^^,^ c ^,^ d ^,^ e ^,^ f ^,^ g ^,^ h
		Run	5	17	14	44	30±2^a^^,^ c ^,^ d ^,^ e ^,^ f ^,^ g ^,^ h
	Old	No Run	4	35	2	21	8±1^a^^,^ b ^,^ d ^,^ f
		Run	6	16	7	35	15±2^a^^,^ b ^,^ c ^,^ e ^,^ g ^,^ h
**Female**	Young	No Run	4	17	3	22	9±1^a^^,^ b ^,^ d ^,^ f
		Run	5	35	5	31	14±1^a^^,^ b ^,^ c ^,^ g ^,^ h
	Old	No Run	3	21	0	14	6±1^a^^,^ b ^,^ d ^,^ f
		Run	3	21	2	27	9±1^a^^,^ b ^,^ d ^,^ f ^,^ g

1 The age of animals in the different groups at the beginning of the 13 week No-Run/Run experiment were: young male/female groups, 3.5 months; old male groups, 15–17 months; old female groups, 15 months.

2 a,b,c,d,e,f,g,h denote significant differences, based on Post-hoc comparisons (Fisher LSD), between the eight experimental groups as follows:

**^a^**difference from the male young sedentary group,

**^b^**difference from the male young exercised group,

**^c^**difference from the male sedentary old group,

**^d^**difference from the male exercised old group,

**^e^**difference indicates difference from the female young sedentary group,

**^f^**difference from the female young exercised group,

**^g^**difference from the female sedentary old group,

**^h^**difference from the female exercised old group.

**Table 2:** Distribution of myofibers into quartiles according to ranges of satellite-cell numbers per myofiber; quartiles are determined based on all myofibers from all experimental groups.

	Quartile number^2^		1st	2nd	3rd	4th	Total no.of myofibers
The range of satellite cell no. per myofiber in each quartile			0–6	7–11	12–20	21–48	(100%)
**Male**	Young	No Run	6%	10%	29%	55%	49
		Run	0%	0%	12%	88%	17
	Old	No Run	31%	40%	26%	3%	35
		Run	0%	38%	50%	13%	16
**Female**	Young	No Run	53%	23%	18%	6%	17
		Run	23%	26%	28%	23%	35
	Old	No Run	71%	24%	5%	0%	21
		Run	33%	43%	24%	0%	21

1 Data presented in this table were collected from the same animals detailed in Table 1.

2 Myofibers from all 8 groups were pooled together and ranked from low to high according to the number of satellite cells per each myofiber. The obtained rank was then divided to four equal quartiles (each containing one quarter of the ranked data). The ranges of satellite-cell numbers contained within each quartile are: 0–6, 1^st^ quartile; 7–11, 2^nd^ quartile; 12–20, 3^rd^ quartile; 21–40, 4^th^ quartile. These ranges are depicted at the top the Table. The total number of myofibers per each group is depicted at the right-hand column (these are same fibers as in Table 1).

**Table 3:** A comparison of the mean number of myogenic and nonmyogenic clones per individual myofiber between the eight experimental groups.^1^

			No. of myofibers analyzed	Mean number of clones per total myofiber analyzed	
				Myogenic	Nonmyogenic
**Male**	Young	No Run	37	2.0	3.6
		Run	37	3.2	3.1
	Old	No Run	32	1.0	4.3
		Run	58	4.4	2.1
**Female**	Young	No Run	31	1.7	2.8
		Run	39	2.5	5.0
	Old	No Run	22	0.8	4.6
		Run	15	1.5	2.0

1 Data presented in this table were collected from the same animals detailed in Table 1.

### Quantification of satellite cells on freshly isolated myofibers

Satellite-cell numbers in individual fast-twitch myofibers from the superficial region of the Gast muscle were determined based on Pax7 immune-detection (Figure 1A). Satellite cell score was compared for all eight groups by means of descriptive and inferential statistics (Figure 1 and Tables 1, 2).

Descriptive statistics on the number of satellite cells in the eight experimental groups is depicted in Table 1. A two-way ANOVA revealed that the number of satellite cells per myofiber significantly differed among groups (between-group factor; F_1,203_ = 23.4, *p* = 0.00001). Regardless of exercise, the number of satellite cells per myofiber was significantly higher in young compared to old groups (within-group factor; F_3,482_ = 68.6, *p*<0.0001 and *p*<0.005) and the number of satellite cells per myofiber was significantly lower in young and old females compared to young and old males (F_1,203_ = 77, *p<0.0001*). Nevertheless, young males manifested the most robust increase in satellite cells per myofiber. Specifically, their minimum number of satellite cells per myofiber was tripled after exercise, and the number of myofibers with more than 41 satellite cells was doubled (Table 1). In both genders and ages, exercise induced a significant increase in the number of satellite cells per myofiber (within-group factor; F_1,203_ = 23.4, *p*<0.0001).

The exercise-induced increase in number of satellite cells per myofiber is also illustrated in cumulative curves of each experimental group (Figure 1B). Each data point shows myofiber percent (out of the total analyzed myofibers) that contained up to and including the number of satellite cells that is specified on the x-axis. These data show that in old male and female rats, exercise increased the abundance of myofibers that contained higher numbers of satellite cells (above median value, which is indicated by gray broken line at the 50% value). This increase was concomitant with decreased abundance of myofibers containing fewer satellite cells (below median value; χ^2^_3_, *p*<0.005). Additionally, these curves show that the age-associated decline in the abundance of myofibers with fewer satellite cells was moderate in females compared to males.

We further analyzed the quartile ranges of satellite-cell number in myofibers. For this, we pooled together the scores of all groups and ranked them from low to high according to the number of satellite cells per individual myofibers. This range was then divided into four equal quartiles. The percent of myofibers of each group in each quartile was extracted (Table 2). As shown, in both ages and in both genders, the 3^rd^ and 4^th^ quartiles contained more myofibers in exercised than in sedentary groups. Specifically, 88% of the myofibers in young exercised males were in the 4^th^ quartile with 21–48 satellite-cells/fiber, compared to only 55% in young sedentary males. Myofibers of old male rats were mostly in the 2^nd^ and 3^rd^ quartiles with 7–20 satellite-cells/myofiber. Notably, while 40% of the myofibers in old sedentary males were in the 2^nd^ quartile, 50% of the myofibers isolated from exercised old males were in the 3^rd^ quartile.

In female rats, the number of satellite cells per myofiber was low compared to males, ranked mainly in the 1^st^ and 2^nd^ quartiles with 0–20 satellite cells (Table 2). About half of the myofibers of young sedentary females contained only 0–6 satellite cells (1^st^ quartile), about 20% in the 2^nd^ and 3^rd^, and just 6% in the 4^th^ quartile. After exercise, however, there was an even distribution of myofibers/quartile (about 25% per quartile) with a conspicuous increase from 6 to 23% in the 4^th^ quartile. About 70% of the myofibers from old sedentary females contained just 0–6 satellite cells (1^st^ quartile), whereas after exercise, 67% of the myofibers were in the 3^rd^ and 4^th^ quartiles, reflecting the exercise-induced increase in the number of satellite-cells/myofiber. Moreover, the number of myofibers with 0–1 satellite cells dropped from 20% in old sedentary females to 0% after exercise. Altogether, these analyses demonstrated that after exercise, the abundance of myofibers with high number of satellite cells increased, along with a decreased abundance of myofibers with fewer satellite cells.

### Clonal analysis of myofiber-associated cells: clone numbers and myogenic vs. nonmyogenic identity

Clonal analyses were performed to determine the impact of aging, of gender and of exercise on stem cells that are tightly associated with Gast myofibers. Clones were defined as myogenic when contained myofibers or when they were positive for Pax7 and MyoD. Clones were either positive or negative for the two markers, which allowed their classification as myogenic or nonmyogenic, respectively, even when myotubes were not apparent.

The mean number of myogenic and nonmyogenic clones per total myofibers is detailed in Table 3, and the mean percent of myogenic clones (out of total clones) is depicted in Figure 3. In the sedentary groups, there was an age-associated decline in abundance of myogenic clones, which was marginal in males and significant in females (Fisher LSD test, MS = 0.15, df = 247, *p* = 0.058 and *p*<0.0005, respectively; Figure 3). These data accord with the age-associated decline in the mean number of satellite cells (Table 1). In old rats (males and females) exercise induced a significant increase in the percent of myogenic clones (two way ANOVA, F _1 247_ = 10.3, *p*<0.005 and *p*<0.0001, males and females, respectively). This effect of exercise on percent of myogenic clones accords with the effect on the mean number of satellite cells (Table 1).

Cumulative curves of the total number of clones (myogenic and nonmyogenic) are depicted in Figure 4A. Each data point shows the percent of myofibers (out of total myofibers) that gave rise to- up to and including the number of clones that is specified on the x-axis. In young and old males and young female rats after exercise, there was a shift toward a greater number of myofibers that give rise to more clones (compared to respective sedentary groups). This conforms the data on the abundance of myogenic clones (Figure 3) and accumulation of more satellite cells per myofiber (Figure 1B) after exercise. Exercising and sedentary females did not differ in the mean number of total clones/myofiber. Nevertheless, the percent of myogenic clones was significantly higher in exercised females. The increased abundance of myogenic clones in the old exercised groups agrees with our finding on exercise-induced increase in Gast mass (Figure S1). Similarly, the decreased post-exercise abundance of nonmyogenic clones (which include cells that differentiate into adipocytes) accords with the decreased percent of fat inside the Gast muscle in the old exercised groups (Figure S1).

The distribution of myogenic clones derived from individual myofibers is presented in Figure 4B. Each data point represents the percent of myofibers (y-axis) that gave rise to a specific number of myogenic clones (x-axis). In males and females, exercise induced a shift toward more myogenic clones. In old rats, at least half the myofibers did not give rise to myogenic clones, while after running 15% and 40% of the myofibers from male and females, respectively, gave rise to 2 or more myogenic clones. The most impressive difference between genders was that regardless of age, myofibers from males gave rise to more clones compared to females. This latter finding is in agreement with the above result that myofibers from males harbor more satellite cells than myofibers from females (Figure 1 and Table 1).

### Body weight, muscle mass and animal locomotion

As detailed above, augmentation of satellite-cell numbers was noted in all the exercised groups. In order to link the characterization of satellite cells to the effect of age and exercise at the “whole muscle” level, we also analyzed changes in total body weight and in mass and fat content of the Gast muscle (Figure S1). Additionally, at the organism level we measured the effect of exercise on spontaneous locomotion in the open-field (Figure S2). {See Material S1 for data, methods and experimental approach}. Our main findings are:

- Exercise had no significant effect on total body weight (Figure S1, panel A). Regardless of exercise or sedentary conditions, young males and females weighed significantly more at the end of the 13-week experiment, indicating that they continued to grow during the experiment. Body weight in the old groups did not change in response to exercise or to the elapsing time of the experiment.
- Exercise did induce significant changes in lean muscle mass in the old groups. Specifically, the ratio between Gast muscle weight and total body weight was significantly higher after exercise in old males and females (Figure S1, panels A,B). In contrast, in the old sedentary rats, this ratio either did not change (males) or significantly declined (females) during the 13-week experiment. Concomitant with the increased Gast weight/total-body-weight ratio in old exercised rats, the percent of intermuscular fat significantly declined (Figure S1, panel C). Taken together, our data suggest that exercise induced a significant increase in lean muscle-mass of old males and females.
- Importantly, the levels of spontaneous locomotion were significantly higher in the exercised vs. sedentary old males (Figure S2). Old exercised females also locomoted more than their sedentary controls, but this difference was not statistically significant (p = 0.06).

It is noteworthy that due to the advanced age and heavier weight the relative intensity of exercise was higher in the old compared to young rats [32], [33]. Yet, our results show that, regardless of the differences in the relative intensity, exercise had a beneficial effect on the number of satellite cells and the number of myogenic clones per myofiber both in the young and in the old rats (see Tables 1 and 3). Nevertheless, in the physiological parameters there was a differential effect of exercise intensity on young compared to old rats. Specifically, exercise induced significant effects in male and female old rats that were manifested in increased Gast muscle-weight/body-weight, reduced muscle adiposity, and increased locomotion. Exercise probably was not stimulatory enough to induce such changes in the young groups.

In summary, exercise running had a persistent beneficial effect on male and female old rats, manifested in increased satellite-cell numbers, abundance of myogenic clones, increased Gast muscle-weight/body-weight, reduced muscle adiposity, and increased locomotion.

## Discussion

We investigated the effects of gender, aging, and moderate-intensity endurance training, on the number and myogenic capacity of satellite cells. Our data demonstrated that in sedentary rats, the number of satellite cells per myofiber declined with age. There was also a reduction in the total number of myofiber-derived clones and in the percent of myogenic clones in old compared to young rats. Exercise, however, ameliorated the aging effects by increasing the number of satellite cells/myofiber in young and old rats. This was accompanied with increased abundance of myogenic clones in old males and females. Changes at the satellite cell level in old rats were accompanied by positive effects on the lean-to-fat content of the Gast muscle and on the levels of spontaneous locomotion. We postulate here that the satellite cell pool size is important for muscle maintenance and therefore, the positive impact of exercise on satellite-cell numbers and properties may have an ameliorating effect in aging.

### Gender and aging effects on satellite cells

Prevalence and severity of sarcopenia differ between genders, starting earlier in females but progressing faster in males [31], [34], [35]. Accordingly, we found an age-associated decline in Gast mass of sedentary rats, which was significant in males and marginal in females. In parallel to the slower rate of muscle loss in females, we show that the reduced satellite-cell numbers in sedentary old rats was more moderate in females. On average, myofibers from old males exhibited a 64% decline in their resident satellite cells, compared to a 34% decline in myofibers from old females. In a recent study we found that female mice displayed a more accelerated age-linked reduction in satellite cells compared to male mice [6]. Such a difference between female rats and mice may be explained by the fact that, at very old age, muscles from females deteriorate more than muscles of old males [36]. Indeed, in our previous experiment [6], female mice were older compared to the present group of old rats. Furthermore, the initial difference in satellite-cell numbers between young male and female mice was lesser than in rats. Hence, an input about satellite-cell numbers from an early time-point in rats may have led to similar conclusions as in mice. Another gender-associated difference was that regardless of age, Gast myofibers from females contained significantly less satellite cells than Gast myofibers from males in both exercise and sedentary conditions. The lower satellite-cell numbers in females accord with our study with mice [6] and may relate to the fact that males have greater muscle-mass than females. These gender-associated differences in satellite cell numbers in rats and mice may be attributed, at least partially, by the differential influences of male and female sex hormones. Both testosterone and estrogen were shown to positively affect satellite cell numbers: testosterone was shown to induce a dose dependent increase in satellite cell numbers, myofiber cross-sectional area, and myofiber nuclei in both young and older men [37], [38], [39] and estrogen supplementation to ovariectomized female rats prior to exercise led to enhanced satellite cell numbers and performance [40], [41]. The age associated decline in estrogen is more prominent in females, where a 90% fall in serum oestradiol occurs across menopause [42], compared to a fairly constant decline in testosterone of 1.6% per year in men that begins in the late third or early fourth decade [43], [44]. Therefore, the differential effects of sex hormones and the different pattern of their decline in aging may contribute to the differences in satellite cell numbers between young and old males and females.

In both mice and rats, an age-associated decline in satellite-cell numbers was noted in fast-twitch (e.g., EDL, Gast) and slow-twitch (Soleus) muscles. In the soleus, the decline was moderate compared to that of the EDL muscle [6], [9], [20], [45]. In humans, results about satellite-cell numbers in aging were less consistent than in rodents: some reported no change [46], [47], others reported a reduction in satellite-cell numbers [48], [49], [50]. It remains debatable whether the age-associated decline in satellite-cell numbers is characteristic to all muscles [14], [51]. Nevertheless, depletion of the satellite cell pool in aging humans was persistently reported [48], [49], [50] when (i) satellite cells were identified based on immunolabeling with markers such as NCAM that allowed inspecting a large sample size of myofibers; and (ii) the group comprised humans at the age of 70 or more, when age-related changes are apparent [52]. Considering the consistency in the reduction of satellite-cell numbers in aging humans and rodents, and in a variety of muscles (legs, arms, mastication) [6], [9], [20], [45], [48], [49], [50], we suggest that this reduction is a general process, not species- or muscle-type- specific.

### Exercise induced an increase in satellite-cell numbers in male and female rats

In this study we analyzed the number of satellite cells situated in myofibers that were isolated from the superficial region of the Gast muscle, a region shown to be composed of fast-twitch fibers, almost exclusively [23], [24], [25]. In order to evaluate the effect of exercise on satellite cell numbers in aging we studied here the most affected myofibers, i.e., the fast-twitch. This was based on a large body of literature showing that in aging there is a preferential loss of fast motor units (especially in non-postural muscles) resulting in a preferentially loss of fast-twitch myofibers [53], [54]. These changes were shown, by others and us, to coincide with a decline in the number of satellite cells which is steeper in fast-twitch than in slow-twitch myofibers [6], [9], [19], [45].

An unequivocal elevation in satellite-cell numbers in old and young rats was achieved after 13-week moderate-intensity running. We show that exercise induced an increase in satellite-cell number-per-myofiber in young and old rats of both genders. This was in concomitance with an exercise induced reduction in the number of myofibers with a few satellite cells at old age. The increase in satellite-cell number-per-myofiber was most remarkable in young male rats. This may be explained, at least partially, by the differential effect of exercise on satellite cells via sex hormones. Specifically, testosterone is a renowned anabolic agent of skeletal muscles, shown to induce muscle hypertrophy in association with increased satellite-cell numbers [55], [56]. Moreover, moderate-intensity endurance training increases testosterone concentrations, probably by its enhanced production [57], [58], [59]. Differently, moderate-intensity endurance exercise does not alter estrogen metabolism [60], [61]. Thus, exercise effect on testosterone may have contributed to the increase in myofibers with greater satellite-cell numbers in young males. It is worthy to note that estrogen was suggested to exert positive effects on satellite cell numbers in the case of straining exercise, such as downhill running, via estrogen receptors mediated mechanisms [40], [41].

Considering the practical implications of satellite-cell pool depletion with age, we identify two cases in which the depleted pool may be significant: (i) when satellite cells are heterogeneous in their ability to produce myogenic progeny and to self-renew; (ii) when each myofiber depends on its own satellite cells. Examination at the single cell level showed that satellite cells are heterogeneous and that there is an age-associated decline in the subpopulation of cells that retains good potency of self-renewal and producing myogenic progeny [6]. To inspect whether a myofiber depends on its own satellite cells, we applied a genetic approach combined with mathematical methods in order to reconstruct lineage trees [62]. This allowed us to reveal that myofibers from intact young and older mice indeed depend on their own satellite cells for maintenance [63]. Altogether, our data imply that the exercise-induced increase in satellite cells numbers/myofiber may support better muscle maintenance upon daily wear.

### Exercise induces augmentation of myogenic clones

The aging environment affects directly or indirectly satellite-cell properties and muscle deterioration [14], [64], [65], [66]. Does the intrinsic potential of satellite cells also change with age? A set of in-vivo and in-vitro experiments showed that muscle regeneration, which is mediated by satellite-cells, depends on environmental influence and on the intrinsic potential of satellite cells [21].

In order to assess the intrinsic properties of individual satellite cells in aging, we calculated the percent of myogenic clones. Our data show the percent of myogenic clones decreased with age, but increased after exercise in old males and females. This exercise-induced increase in myogenic clones implies that the intrinsic satellite-cell properties do not deteriorate with age. This is also in agreement with our finding on the increased Gast mass, in exercised old males and females (Figure S1).

The higher percent of nonmyogenic clones in the old sedentary groups may relate to another age-associated de-regulation, the diversion of some satellite cells to a nonmyogenic fate [14]. Based on in-vitro experiments with cell-lines, freshly isolated myofibers and primary cells, it was suggested that satellite cells can acquire alternative fates such as adipogenic or fibroblastic [14], [67], [68], [69], [70]. Age-associated diversion from a myogenic fate was shown by detecting the expression of Pax7-cre driven reporter in fibroblast-like cells and in myogenic cells that developed in myofiber cultures [14]. Additional studies suggested mesenchymal plasticity of satellite cells based on detecting nonmyogenic cells, including adipocytes, in cultures emanating from single myofibers [69], [71]. Similar to results presented here, nonmyogenic cells were also detected in clonal assays of myofiber-associated cells [11], [71], [72], [73]. It is possible, however, that some or most nonmyogenic cells that develop in myofiber cultures are derived from cells that are in close association with myofibers but not necessarily from Pax7^+^ satellite cells [70], [74], [75], [76].

Enhanced intermuscular adiposity and fibrosis are characteristics of muscle waste as in sarcopenia, myodystrophy or myopathy [65], [77], [78]. It may be that under such conditions, the signaling environment was altered and could no longer support optimal satellite cell performance [14], [79], [80], [81]. As a consequence, satellite cells could divert from the traditional myogenic to an adipogenic/fibrogenic pathway. Alternatively, changes in the signaling milieu may now enhance the proliferation of cells that are in tight association with myofibers and give rise to nonmyogenic progeny [58].

In any event, the exercise-mediated reduced-abundance of nonmyogenic clones is important, since regardless of their origin, accumulation of nonmyogenic instead of myogenic cells that form the contractile units may hinder muscle functioning, maintenance and repair.

### The effect of forced locomotion on spontaneous locomotion

Typically, aging and exercise inversely affect almost all body systems [82], [83], [84]. To achieve a global view on the effect of exercise, the studied rats were tested in the open-field apparatus where they could freely locomote or remain sedentary. Spontaneous locomotion was quantified as an index of their behavior [85], [86], revealing that: (i) The traveled distance significantly decreased in old compared to young sedentary rats (males and females). This result accords with the age-related reduction in locomotor activity that was documented across the animal kingdom [87]. (ii) In old rats, exercise mediated a significant increase of spontaneous locomotion in males. The age-related drop and the exercise-induced increase in locomotion are not necessarily a mere reflection of muscle capacity, but may also reflect changes in the central and peripheral nervous systems [88]. Interestingly, the levels of locomotion significantly correlated with the abundance of myogenic clones, indicating that locomotion level can be used as a predictor for the myogenic properties of satellite cells. Thus, employing a relative simple in-vivo test (the open field) can provide insight into the properties of satellite cells, so that the higher the level of locomotion, the higher the chance that satellite cells produce high percents of myogenic clones.

Conclusions: In this study we demonstrated that exercise induced a considerable reduction in the abundance of myofibers with lower numbers of satellite cells, with a concomitant increase in the abundance of myofibers with a high content of satellite cells. Exercise also induced an increase in the abundance of myogenic clones, suggesting enhanced myogenic performance of the myofiber-associated cells. In old rats, these effects were further associated with enhanced muscle quality (lean-to-muscle composition) and spontaneous activity. We suggest that the boost of satellite-cell numbers and their myogenic performance may represent a possible mechanism by which endurance exercise enhances muscle quality in old age.

## Materials and Methods

### Animals

Male and female Wistar rats of the following age groups were used: Young – 3.5 months, n = 10 per each gender; old males – 15–17 months, n = 9; old females – 15 months, n = 8. Animal age listed throughout this manuscript is based on the age at the beginning of the experiment. Animals were kept under ad-lib nutritional conditions standard rodent chow (Kossoak, 19510), constant temperatures of 22±2°C) and a 14/10 hr light/dark cycle. All animal experiments were reviewed and approved by the Animal Experimental Committee Tel Aviv University Institutional Animal Care and Use Committee, permit number M-06-095.

### Running procedure

Male and female rats were randomly assigned for the running exercise or sedentary group used. We used a motorized low-noise treadmill (running area = 41×114 cm, Horizon ID 100) adjusted to a speed of 0.5 km/h to achieve moderate intensity running [89]. Rats ran 20 minutes a day, 6 days a week, for 13 weeks. A custom-designed Plexiglas mount (treadmill enclosure) divided the belt surface into six compartments, allowing six rats to run simultaneously at the same belt speed [11]. A short video clip of exercise running is available at the supplemented material (Video S1).

Following the 13-week running period, exercised and sedentary rats were sacrificed and their hind-limb Gast muscles were excised for further satellite cell analyzes. Additionally, body weight, Gast muscle mass and levels of spontaneous locomotion were assessed in-vivo for all animals, as described in Material S1.

### Myofiber isolation

Freshly isolated myofibers were used for quantification of their satellite cell content by Pax7 immunostaining and for clonal assays of myofiber associated cells. Intact myofibers were released from the superficial region of the Gast muscle which is enriched with fast-twitch fibers [23], [24], [25]. Myofibers were released after digestion with 0.2% collagenase type I (Sigma-Aldrich) diluted in DMEM (Invitrogen) fortified with antibiotics as previously described [9], [90]. Muscles from young and old rats were digested for 90 or 120 minutes, respectively, for optimal recovery of intact myofibers. Released myofibers were rinsed extensively to eliminate interstitial cells released during the procedure. Myofibers were then dispensed individually into wells pre-coated with Matrigel (BD Biosciences, diluted with DMEM to 1 mg/ml, according to our published procedures [71], [90]). Myofibers were supplemented with growth medium that consisted of DMEM (high glucose, with l-glutamine, 110 mg/L sodium pyruvate, and piridoxine hydrochloride supplemented with 50 U/ml penicillin and 50 mg/ml streptomycin; Invitrogen) supplemented with 20% fetal bovine serum (Biological Industries, Beit Haemek), 10% horse serum (HyClone) and 1% chicken embryo extract (Biological Industries, Beit Haemek). Three hours thereafter myofibers were fixed for subsequent immunostaining analysis to quantify Pax7^+^ cells.

### Single cell cloning

Clonal analyses of myofiber associated cells were performed as we previously described [71]. In brief, well-rinsed myofibers were triturated individually in a tube containing a small volume of medium. The resulting myofiber suspension was dispensed to 24 well trays pre-coated with Matrigel. This procedure which was expected to yield 0–1 satellite cell per well, based on an average number of satellite cells per Gast myofiber of young male rats [11], allowed the progeny of individual progenitors to develop clonally. Wells were tracked from the 2^nd^ day of single cell seeding for 7 days. Half a milliliter of growth medium (0.5 ml) was added after seeding and changed 4 days thereafter. This growth medium promotes both proliferation and spontaneous differentiation as we previously showed [71]. The myogenic identity of each clone was determined based on morphology and expression of muscle specific markers. Clones that contained myotubes were classified as myogenic. Clones that did not develop myotubes within the 7 days of culture were further characterized by double immunostaining for the myogenic markers Pax7 and MyoD. Clones were either positive or negative for the two markers, which allowed us to classify clones as myogenic or nonmyogenic, respectively, even when myotubes were not apparent [9], [11].

### Immunofluorescence

Myofibers and clonal cultures were fixed with 2% paraformaldhyde. Immunostaining and satellite cell quantification were done as we previously described [9], [71]. Primary antibodies were mouse monoclonal: Anti-Pax7 (IgG1, ascites fluid, Developmental Studies Hybridoma Bank; 1∶1000 dilution); anti-MyoD (IgG1, clone 5.8A, BD Biosciences; 1∶400 dilution). When quantifying satellite cells on myofibers, wells were reacted with Pax7 antibody alone. When analyzing clones for myogenic identify, wells were reacted with both anti-Pax7 and anti-MyoD together as the study aimed to detect all myogenic cells regardless of their state of differentiation. The secondary antibody was Cy-3 goat anti-mouse (Jackson Immunoresearch, 1∶500 dilution). Observations were done with an inverted fluorescent microscope (Zeiss, Axiovert200M), controlled by Axiovision4.4 Imaging System. Images were acquired with an AxiocamMRm monochrome CCD camera and composites of digitized images were assembled using Adobe Photoshop software.

### Statistics

All statistical analyses were performed using Statistica 7. Comparisons were carried out with parametric or non-parametric testing depending on whether data conformed to a Gaussian distribution. To describe whether there are significant relationships between several tested variables we employed a correlation test. Analysis of variance was tested either using the parametric MANOVA (multiple analyses of variance) or non parametric Friedman test. When significant differences were found they were followed by post-hoc Fisher LSD test for comparisons. When comparing proportion data (ratio, percents), we carried out the ANOVA on arcsine of square-root-transformed raw data. For comparisons of the distribution of proportional data chi-square tests were performed. For all tests, *P* values less than 0.05 were considered significant.

## Supporting Information

Material S1 Supplemental text.
(0.04 MB DOC)

Figure S1 Total body weight (A), Gast mass/total body weight (B) and fat content of the Gast muscle (C) of young and old male/female exercised/sedentary rats, before (open bars) and after (gray bars) 3 months running exercise. Gast mass and its fat content were measured by DEXA. To assess the fat content of the Gast muscle, a predetermined constant muscle tissue volume from the largest diameter of the muscle was measured, in order to reflect mere changes in the relative density of the muscle and exclude growth dependent changes in the mass of the whole muscle over the 3 months of the exercise session. Each bar depicts average values and error bars indicate SEM values.
(4.53 MB TIF)

Figure S2 Total distance travelled over 15 minutes in the open-field arena, by male and female, young and old sedentary (gray bars) and exercised (open bars) rats. Each bar depicts average values and the error bars indicate the standard error of the mean (SEM). Insets at the top of the figure represent the actual travelled trajectories of an old exercised (gray shaded) and an old sedentary (without shading) male.
(4.53 MB TIF)

Video S1 Rats running on a treadmill.
(2.02 MB MP4)
