# Supplementary material for: Reduced Satellite Cell Numbers and Myogenic Capacity in Aging Can Be Alleviated by Endurance Exercise
Source: PLoS One. 2010 Oct 12;5(10):e13307. doi: 10.1371/journal.pone.0013307 (PMC2953499; doi:10.1371/journal.pone.0013307)
Supplement: Material S1 — Supplemental text. (0.04 MB DOC) [file pone.0013307.s001.doc]

**Supplemented material**

***Body weight, muscle mass and fat content*** (Figure S1):

The Gast muscle mass and fat content were measured before and after the 13 weeks of exercise period to further study the effects of exercise on the gross anatomy of the muscle from which we investigated satellite cell properties. Quantification of the Gast mass and its fat content were achieved by The PIXImus II Densitometer (Lunar, Madison, WI) which utilizes Dual Energy X-ray Absorptiometry (DEXA) technology. DEXA machine was used with the advantage of measuring tissue composition in the living animal, enabling follow up on the same individual before and after three months of exercise. This Densitometer is commonly used to measure bone densitometry and body composition of mice and other small animals [76,77]. For the duration of measurement, animals were anesthetized with isofluorane (Rhodic) using an anesthetic machine (Ohmeda, Ohio) with medical-grade oxygen (2-3% vol.). The Gast boundaries were defined by the user based on the visual outlines of the muscle. Measurements of the percent of fat in the Gast tissue were done on a fixed-sized sample placed at the largest diameter of the Gast muscle. This was done in order to eliminate the impact of whole muscle growth (which reflect the effect of developmental processes rather than exercise effect, especially in the young animals).

*Total body weight* (Figure S1, panel A): We found that exercise did not have a significant effect on total body weight since regardless of exercise, the total body weight: (a) of young and old males was significantly higher than of young and old females (Two way ANOVA with repeated measures F _1, 29_ = 14.5, *p* < 0.001); (b) of young male and female rats significantly increased during the 3 months of the experiment (F _1, 29_ = 7.2, *p* < 0.05) probably reflecting the effect of growth. The body weight of old animals, however, did not change probably since growth has already reached a plateau at a younger age.

*Gast weight/Total body weight and fat content* (Figure S1, panels B and C): At young age of both genders, following the 13 weeks of the experiment, there was a significant decline in ratio of the Gast weight per body weight (two way ANOVA with repeated measures, F _1, 29_ = 5.1, *p* < 0.05) which was not reversed by exercise. This decline can be explained by the fact that animals are still growing (Figure S1 A) and the rate of whole animal growth is higher than the rate of the Gast muscle growth induced by exercise. At old age, however, when male and female animals have ceased growing the effect of exercise was evident (two way ANOVA with repeated measures, F _1, 61_ = 5.9, F _1, 61_ =12.36, respectively, *p* < 0.05, Figure S1 B) and stemmed from an increase of lean muscle since the percent of intermuscular fat was significantly reduced (Two way ANOVA with repetitions, F _1, 64_ = 4.6, *p* < 0.05, Figure S1 C) in old exercised rats. These results accord with the increased numbers of satellite cells, abundance of myogenic clones and reduction of nonmyogenic clones (that include adipocytes). Taken together our data suggest that exercise ameliorated the negative effects inflicted by a sedentary routine on the Gast muscles.

***Levels of spontaneous activity*** (Figure S2):

We used the open-field test to assess the effects of age and exercise on spontaneous activity, which is known to decline in aging rodents and humans [78], perhaps due to loss of muscle mass. In the ontogeny of various rodent species, each with different motor capacities, spontaneous activity in the open-field test was found to correlate with Gast muscle competence [79]. We therefore opted here to test if levels of spontaneous locomotion correlated with the tested parameters of gross muscle physiology and satellite cell properties. A 2x2 m open field arena was constructed by enclosing a tiled floor with plywood planks (60 cm high). The testing room was air-conditioned and illuminated with two 300W light bulbs directed to the white ceiling in order to provide diffused illumination of the arena. Spontaneous locomotion of the animals was detected by a video camera (Ikegami B/W ICD-47E) which was positioned above the arena and provided a top view of the animal. Camera was linked to a computerized tracking system and image analyzer (Ethovision by Noldus, NL) that acquired data about distance traveled (overall distance traveled during the 15-min observation). Rats were placed individually in the open field, and the distance they spontaneously travelled during 15 minutes was measured as previously described [80].

We found that spontaneous locomotion of old exercising male rats was significantly higher compared with old sedentary rats (Non parametric Friedmann test, χ^2^_3_= 87.4, *p* < 0.005; Figure 2). A trend of elevating locomotion levels after exercise was also noted in the group of old females. The lack of statistical significance may stem from the smaller group of old exercising female rats.

Spontaneous activity significantly correlated with the abundance of myogenic clones (r² = 0.24, *p* < 0.05), so that the higher the level of locomotion the higher percent of myogenic clones. This suggests that after exercise the level of locomotion can be used as a predictor of the myogenic properties of satellite cells. Levels of activity also well accorded with the effect of exercise on the Gast muscle mass and its fat content. Altogether, the present results suggest a direct link between the increase spontaneous locomotion induced by exercise and improved muscle properties.
