# Supplementary figures and images for: Reduced Satellite Cell Numbers and Myogenic Capacity in Aging Can Be Alleviated by Endurance Exercise
Source: PLoS One. 2010 Oct 12;5(10):e13307. doi: 10.1371/journal.pone.0013307 (PMC2953499; doi:10.1371/journal.pone.0013307)

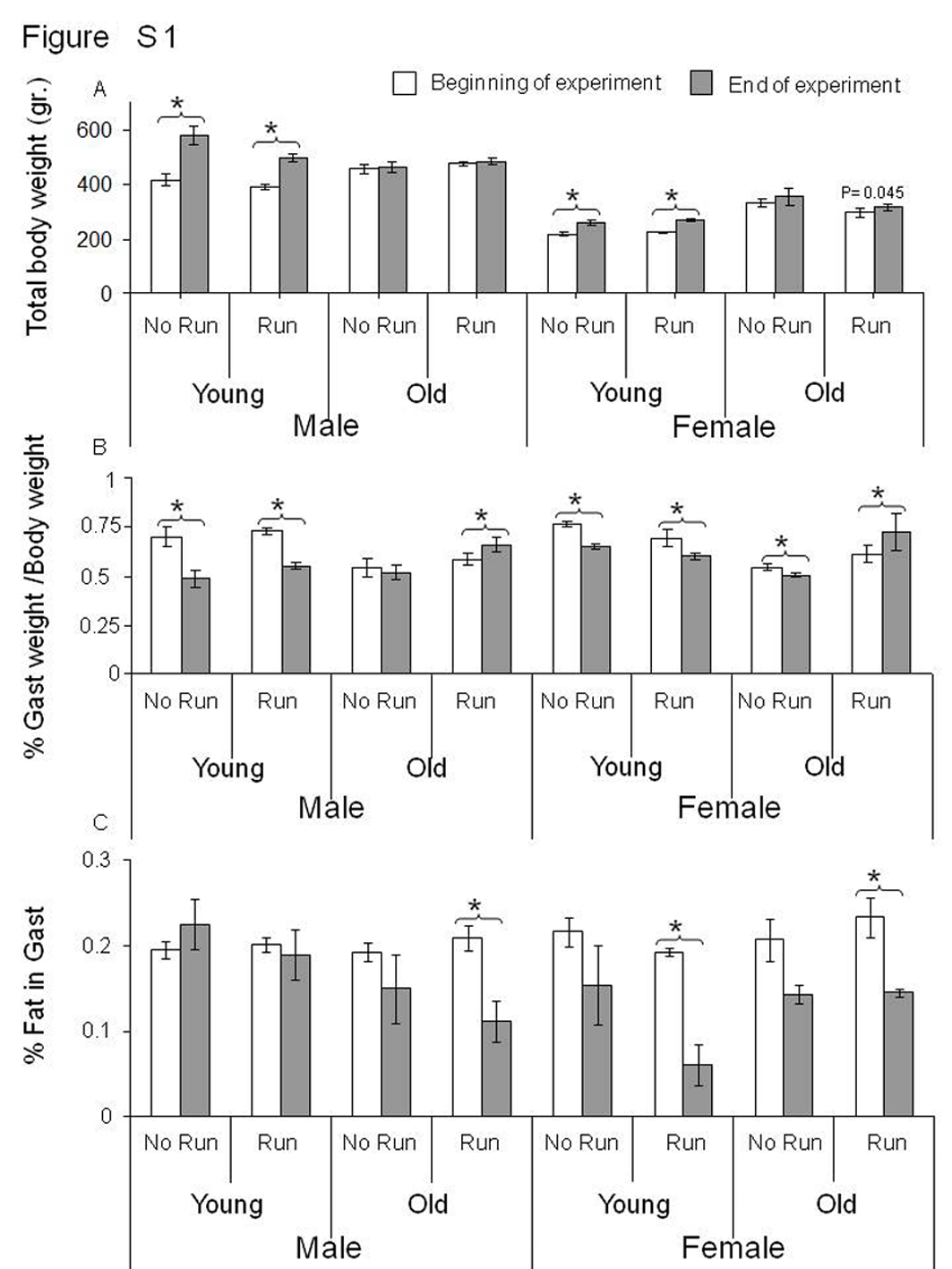

Supplement: Figure S1 — Total body weight (A), Gast mass/total body weight (B) and fat content of the Gast muscle (C) of young and old male/female exercised/sedentary rats, before (open bars) and after (gray bars) 3 months running exercise. Gast mass and its fat content were measured by DEXA. To assess the fat content of the Gast muscle, a predetermined constant muscle tissue volume from the largest diameter of the muscle was measured, in order to reflect mere changes in the relative density of the muscle and exclude growth dependent changes in the mass of the whole muscle over the 3 months of the exercise session. Each bar depicts average values and error bars indicate SEM values. (4.53 MB TIF) [file pone.0013307.s002.tif]

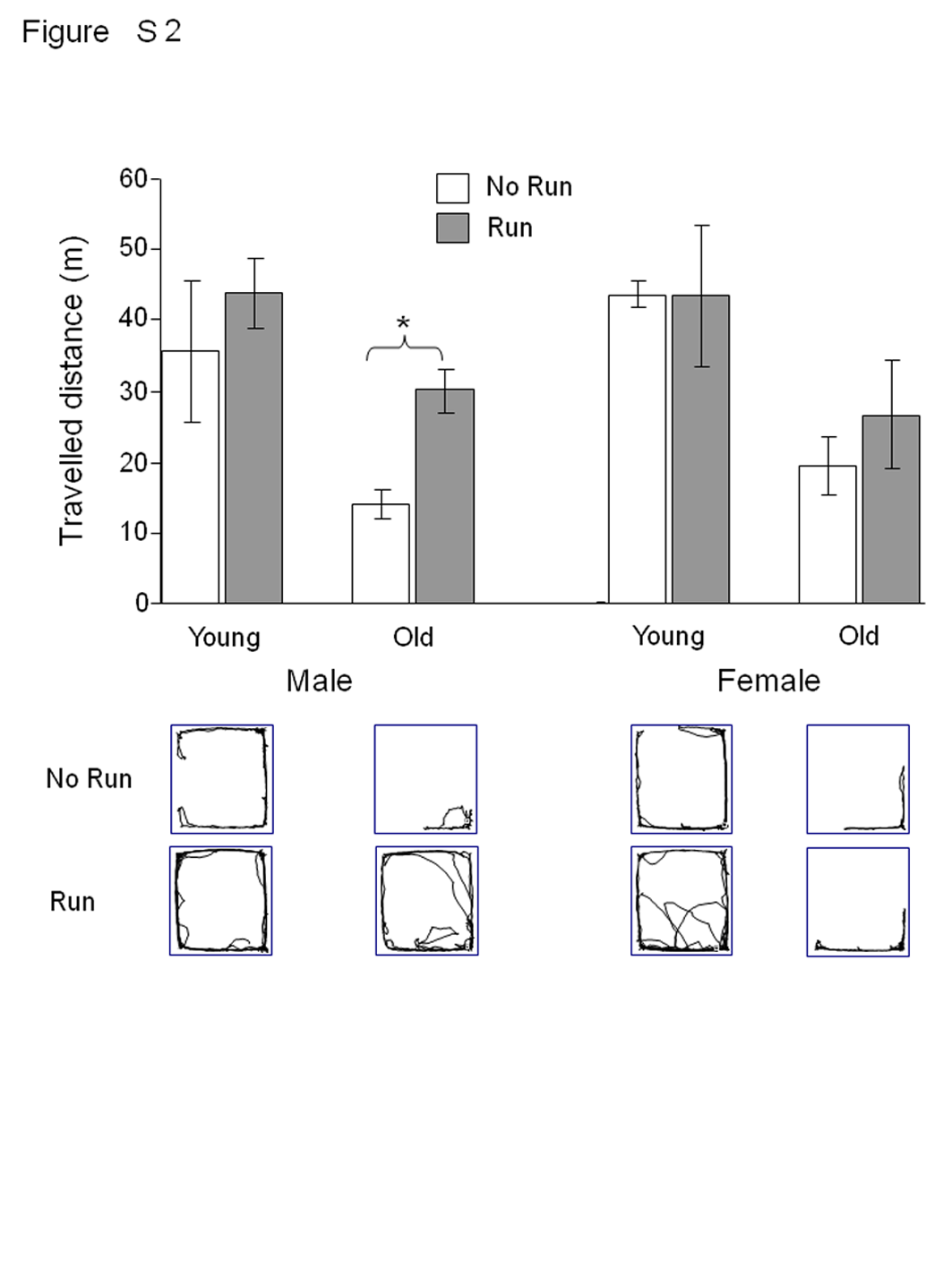

Supplement: Figure S2 — Total distance travelled over 15 minutes in the open-field arena, by male and female, young and old sedentary (gray bars) and exercised (open bars) rats. Each bar depicts average values and the error bars indicate the standard error of the mean (SEM). Insets at the top of the figure represent the actual travelled trajectories of an old exercised (gray shaded) and an old sedentary (without shading) male. (4.53 MB TIF) [file pone.0013307.s003.tif]
